# Health outcomes and healthcare utilization associated with four undiagnosed chronic conditions: evidence from nationally representative survey data in Sri Lanka

**DOI:** 10.1186/s44263-024-00075-0

**Published:** 2024-07-08

**Authors:** Nilmini Wijemunige, Pieter van Baal, Ravindra P. Rannan-Eliya, Owen O’Donnell

**Affiliations:** 1 Institute for Health Policy, Colombo, Sri Lanka; 2 Erasmus School of Health Policy & Management, Erasmus University Rotterdam, Rotterdam, The Netherlands; 3 Erasmus School of Economics, Erasmus University Rotterdam, Rotterdam, The Netherlands

**Keywords:** Non-communicable disease, Undiagnosed, Coronary heart disease, Hypertension, Diabetes, Depression

## Abstract

**Background:**

Low awareness of chronic conditions raises the risk of poorer health outcomes and may result in healthcare utilization and spending in response to symptoms of undiagnosed conditions. Little evidence exists, particularly from lower-middle-income countries, on the health and healthcare use of undiagnosed people with an indication of a condition. This study aimed to compare health (physical, mental, and health-related quality of life (HRQoL)) and healthcare (inpatient and outpatient visits and out-of-pocket (OOP) medical spending) outcomes of undiagnosed Sri Lankans with an indication of coronary heart disease (CHD), hypertension, diabetes, and depression with the outcomes of their compatriots who were diagnosed or had no indication of these conditions.

**Methods:**

This study used a nationally representative survey of Sri Lankan adults to identify people with an indication of CHD, hypertension, diabetes, or depression, and ascertain if they were diagnosed. Outcomes were self-reported measures of physical and mental functioning (12-Item Short Form Survey (SF-12)), HRQoL (EQ-5D-5L), inpatient and outpatient visits, and OOP spending. For each condition, we estimated the mean of each outcome for respondents with (a) no indication, (b) an indication without diagnosis, and (c) a diagnosis. We adjusted the group differences in these means for socio-demographic covariates using ordinary least squares (OLS) regression for physical and mental function, Tobit regression for HRQoL, and a generalized linear model (GLM) for healthcare visits and OOP spending.

**Results:**

An indication of each of CHD and depression, which are typically symptomatic, was associated with a lower adjusted mean of physical (CHD -2.65, 95% CI -3.66, -1.63; depression -5.78, 95% CI -6.91, -4.64) and mental functioning (CHD -2.25, 95% CI -3.38, -1.12; depression -6.70, 95% CI -7.97, -5.43) and, for CHD, more annual outpatient visits (2.13, 95% CI 0.81, 3.44) compared with no indication of the respective condition. There were no such differences for indications of hypertension and diabetes, which are often asymptomatic.

**Conclusions:**

Living with undiagnosed CHD and depression was associated with worse health and, for CHD, greater utilization of healthcare. Diagnosis and management of these symptomatic conditions can potentially improve health partly through substitution of effective healthcare for that which primarily responds to symptoms.

**Supplementary Information:**

The online version contains supplementary material available at 10.1186/s44263-024-00075-0.

## Background

The burden of non-communicable diseases (NCDs) is large and growing in low- and middle-income countries (LMICs) [1]. In Sri Lanka, cardiovascular and metabolic diseases account for more than a quarter of the disease burden, compared to less than one-fifth in high-income countries [2].

A large proportion of people living with NCDs or their risk factors are undiagnosed [3–6]. In South Asia, around two-thirds of men with hypertension are undiagnosed, compared to 31% in high-income, Western countries [3]. Over half of diabetics in LMICs are undiagnosed [4]. In Sri Lanka, 47% and 38% of people with indications of hypertension and diabetes, respectively, are undiagnosed [7, 8]. There is also substantial underdiagnosis of CHD [9–11], and likely underdiagnosis of depression [12], a condition with a bidirectional relationship with CHD [13], diabetes [14], and their risk factors [15] in LMICs.

People in the early stages of developing a chronic condition are often asymptomatic, particularly for hypertension and diabetes. When symptoms do emerge, treatment may primarily respond to the symptoms without managing the underlying, still undiagnosed, condition [16–21]. If undiagnosed people with indications of chronic conditions experience worse health, make greater use of healthcare, and incur more OOP spending, then there may be potential for earlier diagnosis and management not only to slow or prevent disease onset but also to improve health immediately and reduce pressures on health systems and household finances.

There is little evidence from LMICs to determine whether people with an indication but not a diagnosis of a chronic health condition do experience worse health and make greater use of healthcare. An Indonesian study found that people with an indication of diabetes or hypertension that was undiagnosed did not have significantly higher healthcare utilization and expenditures, but undiagnosed people with an indication of a heart problem were more frequent users of outpatient care than people without these conditions [22]. In China, physical and mental functioning of people with an indication of hypertension without diagnosis were similar to those of people with no indication [23]. Even for high-income countries, there is limited evidence on health and healthcare utilization associated with having an indication of a chronic condition without a diagnosis. One small-scale study in Finland found that the physical functioning of people with an indication of hypertension without diagnosis was lower than that of people without hypertension [24]. A subnational study in Japan found that people with undiagnosed depression had lower physical and mental functioning, HRQoL, and more healthcare utilization than people without depression [25]. Each of these studies focused on a limited set of outcomes of one or two chronic conditions.

Using nationally representative data from Sri Lanka, this study aimed to add to the limited evidence from LMICs on the association between having an indication without diagnosis of each of four major chronic conditions—CHD, hypertension, diabetes, and depression—and both health (physical, mental, and HRQoL) and healthcare (inpatient and outpatient utilization, and OOP medical spending) outcomes.

## Methods

We used data from the Sri Lanka Health and Ageing Study (SLHAS) conducted from 9 November 2018 to 14 November 2019 [26]. A multi-stage cluster random sampling design, stratified by district, residential sector, and area socioeconomic status (SES) was used to collect data in 297 sampling units selected by probability-proportionate-to-size sampling in all districts of Sri Lanka [27]. Within each sampling unit (smallest administrative division), households were randomly selected, and one adult (18 years and older) was randomly chosen from each household roster, with oversampling of those aged ≥ 70 years. After the application of sampling weights, the sample was representative of the adult population of Sri Lanka in 2019 by gender, age, geographical region, area SES, and ethnicity [27].

Data were collected using a computer-assisted personal interviewing platform, iFormBuilder (Zerion Software Inc., Herndon, VA, USA), with built-in skip logic and checks for unlikely values during data entry. During data cleaning, less likely values were cross-checked using manually recorded clinic checklists.

### Identification of chronic conditions

In addition to completing a questionnaire, each respondent was asked to bring their medical records to the interview and to give consent for the enumerator to consult these records. An inventory of each respondent’s medicines was taken. Blood pressure, weight, and height were measured, and a blood sample was taken. Blood pressure was measured two times, 10 min apart, on one occasion using an OMRON HEM-7320 blood pressure monitor (OMRON Healthcare Co., Ltd., Kyoto, Japan) by a trained enumerator following standard procedure [7]. We used the mean of the two measurements. Participants were asked to fast for 12 h prior to attending the clinic for data collection. A venous blood sample was taken from all consenting participants, and those who provided an initial fasting sample underwent an oral glucose tolerance test [8]. Samples were stored within 6–10 h of initial collection in a field freezer (TwinBird Freezer SC-DF25, Twinbird Corporation, Niigata, Japan, and Glacio 55L Portable Cooler Fridge PFN-E-WEA-L-GR, New Aim Pty Ltd, Melbourne, Australia) at − 40 to − 20 °C for transport to the Sri Lanka Medical Research Institute (Colombo) for testing.

For each of four chronic conditions—CHD, hypertension, diabetes, and depression—we distinguished between respondents who (a) had been diagnosed (*diagnosed*), (b) showed an indication but had not been diagnosed (*indicated*), and (c) showed no indication and had not been diagnosed (*no condition*). A respondent was defined as diagnosed with CHD if they reported ever being diagnosed with angina, myocardial infarction or coronary artery disease, or their medical records indicated such a diagnosis. They were defined as indicated for CHD if they were not diagnosed but they satisfied the criteria on the Rose angina questionnaire of ever having chest pain that appeared upon exertion, was situated at any level of the sternum or left anterolateral chest and arm, which caused the respondent to slow down or stop while walking, and was relieved within ten minutes of rest, or they reported ever having severe chest pain across the front of the chest for thirty minutes or more [28, 29]. The Rose angina questionnaire is a standardized tool for detecting angina based on self-reported symptoms that has been used in a wide range of research and clinical settings [11, 28] and has been validated for use in Sri Lanka [30].

A respondent was categorized as diagnosed with hypertension if they reported a diagnosis or their medical records showed this, or they reported taking antihypertensives in the past 14 days. They were defined as indicated for hypertension if they were not diagnosed and had a systolic blood pressure of at least 140 mmHg or a diastolic blood pressure of at least 90 mmHg.

A respondent was identified as diagnosed with diabetes if they reported a diagnosis or their medical records showed this, or they reported taking oral hypoglycaemics or insulin in the past 14 days. They were defined as indicated for diabetes if they were not diagnosed but had a fasting plasma sugar of 126 mg/dL or more, or a random (6 participants had not fasted) or oral glucose tolerance test showed a plasma sugar of 200 mg/dL or more [8].

A respondent was categorized as diagnosed with depression if they reported a diagnosis or their medical records showed this. They were indicated for depression if it had not been diagnosed but they scored 10 or more on the Patient Health Questionnaire (PHQ-9) questionnaire [31]. A score ≥ 10 maximizes sensitivity and specificity for major depression [32] and is the threshold validated and used in Sri Lanka [31].

### Outcomes

We used the physical component score (PCS) and mental component score (MCS) from the SF-12 questionnaire [33] to measure physical and mental health functioning, respectively. Both scores were calculated for each respondent using an algorithm developed from a sample in the USA, where scores were standardized to give a mean score of 50 and a standard deviation of 10 [33]. Scores below 50 indicate poorer function.

For each respondent, HRQoL was obtained from their responses to the EQ-5D-5L questionnaire [34]. The EQ-5D-5L is a multi-attribute utility instrument used to measure health status in five domains (mobility, self-care, usual activities, pain and discomfort, anxiety, and depression) with five levels of severity ranging from “no problems” to “extreme/unable”. Each combination of responses was mapped to a utility value using tariffs derived from Sri Lankan data [35], where 1 represents “perfect health” and 0 represents death, and where negative values (states worse than death) are possible.

We measured healthcare utilization with self-reported inpatient stays, which included any admission to a bed in a public or private hospital, and outpatient visits. Outpatient visits covered any visit to a facility that did not require admission or an overnight stay, and included public and private specialist and general clinics, allied health visits (e.g., to see a physiotherapist or dietician), public health clinics, such as medical officer of health and midwife clinics, visits to a pharmacy, laboratory or imaging center, and non-Western medicine clinics. The SLHAS randomly varied recall periods across participants in order to analyze the impact of recall periods on reported healthcare utilization in a study separate from this one. They were 1, 6, and 12 months (22%, 18%, and 60% of the sample) for inpatient visits and 7, 14, and 28 days (20%, 20%, and 60% of the sample) for outpatient visits. Data on the number of inpatient and outpatient encounters over a specified recall period were annualized.

Self-reported OOP spending for direct medical costs, including hospitalization and consultation fees, medicine and medical supplies, investigations, and informal payments were annualized. Amounts were converted to US dollars using the average US dollar exchange rate for the month the respondent was interviewed, as reported by the Central Bank of Sri Lanka, as an appropriate way to handle the devaluation of the local currency [36].

### Covariates

Socio-demographic characteristics included age in 10-year age groups (18–29, 30–39, 40–49, 50–59, 60–69, 70–79, 80 +), sex (male, female), ethnicity (Sinhala, Tamil, Muslim/Moor/Malay, Other), education (no formal, primary, secondary, and tertiary), sector (urban, rural, estate, rural/estate) and province of residence, SES, household size (number of persons), and proportions of household members aged under 15 years and above 60 years. SES was proxied by quintile groups of an index obtained as the first principal component of an analysis of household assets, water and sanitation facilities, housing quality, and other assets [37] (Additional file 1: Supplementary methods). Standard body mass index (BMI) categories (normal < 25 kg/m^2^, overweight 25–29.9 kg/m^2^, obese ≥ 30 kg/m^2^), which were used in the sensitivity analysis, were calculated based on the weight in kilograms measured by an OMRON BF511 Body Composition Monitor (OMRON Healthcare Co., Ltd., Kyoto, Japan) and height measured in centimeters by a seca 240 mechanical measuring rod (stadiometer) (seca, Hamburg, Germany).

### Statistical analysis

We estimated the mean of each outcome for each group defined as having no condition, being indicated, and being diagnosed for each of the four chronic conditions (CHD, hypertension, diabetes, and depression). We used a z-test to test the null of no difference in the means for indicated vs no condition, and diagnosed vs no condition. We used multivariate regression to estimate differences in the mean of each outcome between the three groups (no condition, indicated, and diagnosed) adjusted for the covariates (with age groups interacted with sex). For regressions of PCS and MCS scores, which are normally distributed, we used OLS. For HRQoL, following much analysis [38–40], we assumed a Tobit model to account for censoring at 1 that arose from anchoring EQ-5D-based utility values at that value for “full health”, and used maximum likelihood estimation. For regressions of inpatient and outpatient visits, which are counts data, and OOP spending, which are skewed data with many zero values, we used a GLM with a Poisson distribution, a log link, and robust standard errors. Correct specification of the conditional mean is sufficient for the consistency of this pseudo-maximum-likelihood estimator [41], which performs well with many zeros [40] and is often used to model medical spending data [41]. We present estimates from these models of average marginal effects (AME): the change in the mean of the outcome associated with a unit change in an independent variable that is estimated for each observation and averaged over the sample. We repeated the multivariate regressions extended to include BMI, which is usually positively associated with higher risk of CHD, hypertension, and diabetes [42, 43] but can also be associated with poorer health outcomes after controlling for these and other conditions [44]. In all analyses, we applied sample weights and estimated robust standard errors adjusted for sample stratification and clustering. A *p*-value less than 0.05 was considered statistically significant.

Data were missing for PCS, MCS and HRQoL (< 5%), inpatient and outpatient visits (< 3.5%), OOP spending (< 3%), and covariates (< 1%). To avoid selection bias that may result if participants with missing data were excluded, we assumed that data were missing at random and imputed them using multiple imputations with chained equations and predictive mean matching using 10 nearest neighbors (Additional file 1: Supplementary methods). As a sensitivity analysis, we also performed a complete case analysis instead of using multiply-imputed data. All analyses were performed using Stata 17.0 [45].

## Results

Table 1 shows characteristics of the analysis sample after imputation. The mean age was 50.1 years (standard deviation (SD) 17.2) and 51% were female. A majority (82.1%) had secondary education or above. More than half (54.9%) of the sample were in the rural sector. The average household size was 2.98 (SD 1.4), with the proportion of household members aged above 60 years and below 15 years being 0.23 (SD 0.33) and 0.07 (SD 0.16), respectively, on average. Characteristics of the complete cases sample were very similar (Additional file 1: Table S1).

**Table 1:** Sample characteristics, *n* = 6665

	*n* / mean	% / SD
Age, mean (SD)	50.1	17.2
Sex		
Male	3268	49.0
Female	3397	51.0
Ethnicity		
Sinhala	4707	70.6
Tamil	1504	22.6
Muslim	428	6.4
Other	26	0.4
Education		
No formal schooling	258	3.9
Primary educated	937	14.1
Secondary educated	5199	78.0
Tertiary educated	272	4.1
Sector		
Urban	2024	30.4
Rural	3661	54.9
Estate	170	2.6
Rural/Estate	810	12.2
Province		
Western	1435	21.5
Central	976	14.6
Southern	851	12.8
Northern	691	10.4
Eastern	553	8.3
North-Western	548	8.2
North-Central	477	7.2
Uva	467	7.0
Sabaragamuwa	667	10.0
SES quintile		
Poorest	1568	23.5
Poorer	1328	19.9
Middle	1245	18.7
Richer	1220	18.3
Richest	1304	19.6
Household size, mean (SD)	2.98	1.4
Proportion below 15, mean (SD)	0.07	0.16
Proportion above 60, mean (SD)	0.23	0.33

Columns show *n* (%) unless specified as mean (SD). Sample weights not applied. *SES* is socioeconomic status, *SD* is standard deviation. The percentages for SES quintile groups are not 20% because the groups were constructed to account for 20% after the application of sample weights

Table 2 shows, for each of the four chronic conditions, estimates of the population percentages with an indication but no diagnosis (indicated), a diagnosis (diagnosed), and with neither an indication nor diagnosis (no condition). We estimated that 5.7% (95% CI 5.0, 6.5) of the adult population of Sri Lanka had an indication of CHD but had not been diagnosed and 3.9% (95% CI 3.4, 4.4) had been diagnosed with CHD. Meanwhile, 13.0% (95% CI 11.8, 14.2) of the population had an indication of hypertension with no diagnosis, and 16.7% (95% CI 15.5, 17.8) had diagnosed hypertension. For diabetes, 7.2% (95% CI 6.4, 8.0) of the population had an indication of diabetes, and 13.6% (95% CI 12.3, 14.8) had a diagnosis. We estimated that 4.3% (95% CI 3.6%, 4.9%) of the population had an indication of depression and only 1.0% (0.7%, 1.3%) of the population had a diagnosis.

**Table 2:** Estimated prevalence of chronic conditions by indication and diagnosis

	*n*	% (95% CI)
CHD		
No condition	5896	90.4 (89.4, 91.3)
Indicated	382	5.7 (5.0, 6.5)
Diagnosed	387	3.9 (3.4, 4.4)
Hypertension		
No condition	4132	70.3 (68.7, 71.9)
Indicated	975	13.0 (11.8, 14.2)
Diagnosed	1558	16.7 (15.5, 17.8)
Diabetes		
No condition	5054	79.2 (77.8, 80.6)
Indicated	499	7.2 (6.4, 8.0)
Diagnosed	1112	13.6 (12.3, 14.8)
Depression		
No condition	6224	94.8 (94.1, 95.5)
Indicated	377	4.3 (3.6, 4.9)
Diagnosed	64	1.0 (0.7, 1.3)

Imputed data used (*N* = 6665). Sample weights applied for percentage and confidence intervals (CI)

There was substantial multimorbidity (Additional file 1: Table S2). For example, we estimated that among those who had an indication or diagnosis of CHD, 52.0% (95% CI 47.0, 57.1) also had an indication or diagnosis of hypertension and 32.7% (95% CI 27.6, 37.7) had an indication or diagnosis of diabetes. Of those with an indication or diagnosis of depression, we estimated that 42.2% (95% CI 35.0, 49.4) had an indication/diagnosis of hypertension, 32.5% (95% CI 25.7, 39.4) had an indication/diagnosis of diabetes, and 22.3% (95% CI 16.6, 27.9) had an indication/diagnosis of CHD. There was also a substantial overlap between hypertension and diabetes.

**Fig. 1:**
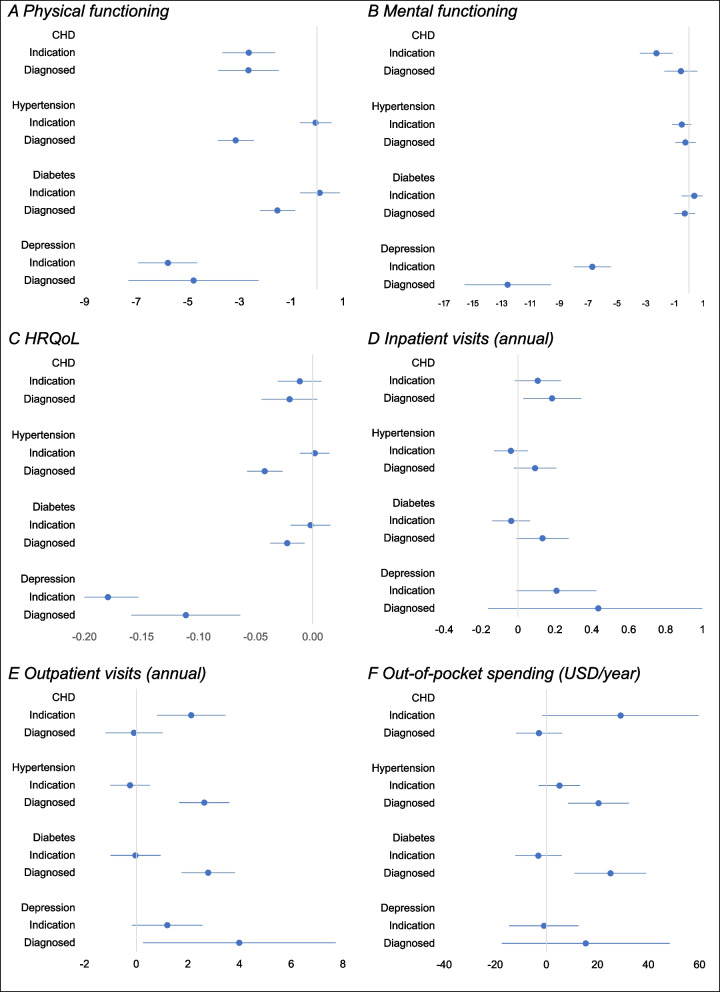
Adjusted differences in mean health and healthcare outcomes between indication or diagnosis and absence of each chronic condition

Table 3 shows the estimated mean of each outcome by category of each chronic condition, and Fig. 1 shows the estimated adjusted difference in means between those indicated with each condition and those without that condition as well as the respective difference between those diagnosed with each condition and those without that condition (point estimates in Additional file 1: Table S3). Without and with adjustment, the mean PCS scores with an indication and with a diagnosis of CHD were lower—indicating lower physical functioning—than the mean score of those without any indication or diagnosis of CHD. After adjustment, the mean PCS score of those with an indication of CHD was 2.7 points (95% CI 1.6, 3.7) lower than the mean score of those without CHD. The mean difference between those diagnosed with CHD and those without the condition was the same (2.7, 95% CI 1.5, 3.8). Having an indication of CHD was associated with an adjusted mean MCS score that was 2.3 points (95% CI 1.1, 3.4) lower (worse mental functioning) than the respective score without CHD. Compared with not having CHD, a diagnosis of that condition was not associated with any difference in mental functioning even before adjusting for covariates. Point estimates show that respondents with an indication of CHD had lower mental functioning than those diagnosed with the condition, although the 95% CIs of the adjusted mean differences overlap. Compared with not having CHD, mean HRQoL was lower among those with an indication of CHD, and lower still for those with a diagnosis, with only the difference for those with a diagnosis statistically significant at the 0.05 level.

**Table 3:** Mean health and healthcare outcomes by indication and diagnosis of chronic conditions

	**Health functioning (SF-12)**		**HRQoL (95% CI)**	**Inpatient visits (95% CI)**	**Outpatient visits (95% CI)**	**OOP spending, USD (95% CI)**
	**Physical (95% CI)**	**Mental (95% CI)**				
All	48.93 (48.60, 49.25)	50.39 (50.05, 50.73)	0.87 (0.86, 0.87)	0.23 (0.19, 0.27)	4.95 (4.52, 5.37)	17.52 (14.50, 20.54)
CHD						
No condition	49.44 (49.12, 49.76)	50.57 (50.23, 50.92)	0.87 (0.87, 0.88)	0.21 (0.17, 0.25)	4.67 (4.24, 5.11)	15.70 (12.63, 18.78)
Indicated	45.88 (44.52, 47.24)***	47.44 (46.00, 48.88)***	0.83 (0.81, 0.86)**	0.24 (0.17, 0.32)	7.85 (5.77, 9.93)***	35.15 (16.68, 53.62)**
Diagnosed	41.37 (39.58, 43.16)***	50.54 (48.86, 52.21)	0.75 (0.71, 0.79)***	0.61 (0.38, 0.84)***	6.98 (5.35, 8.61)**	33.76 (15.81, 51.70)**
Hypertension						
No condition	50.44 (50.13, 50.75)	50.37 (49.97, 50.78)	0.89 (0.89, 0.90)	0.20 (0.15, 0.25)	4.26 (3.77, 4.75)	12.09 (9.02, 15.15)
Indicated	48.77 (48.01, 49.53)***	50.58 (49.79, 51.36)	0.86 (0.84, 0.88)***	0.17 (0.11, 0.23)	4.27 (3.33, 5.20)	17.33 (8.42, 26.24)
Diagnosed	42.66 (41.86, 43.46)***	50.33 (49.64, 51.01)	0.76 (0.74, 0.78)***	0.39 (0.29, 0.50)***	8.35 (7.32, 9.38)***	40.59 (28.13, 53.06)***
Diabetes						
No condition	49.81 (49.50, 50.12)	50.37 (50.03, 50.72)	0.88 (0.88, 0.89)	0.19 (0.16, 0.22)	4.37 (3.94, 4.79)	13.32 (10.41, 16.23)
Indicated	48.39 (47.32, 49.46)*	50.77 (49.85, 51.69)	0.85 (0.83, 0.87)**	0.17 (0.08, 0.25)	4.48 (3.27, 5.69)	13.97 (1.86, 26.07)
Diagnosed	44.03 (43.15, 44.91)***	50.29 (49.38, 51.19)	0.78 (0.76, 0.80)***	0.47 (0.23, 0.71)**	8.58 (7.19, 9.96)***	43.97 (29.22, 58.71)***
Depression						
No condition	49.36 (49.06, 49.66)	50.81 (50.46, 51.17)	0.88 (0.87, 0.89)	0.20 (0.17, 0.24)	4.82 (4.40, 5.24)	16.72 (13.85, 19.60)
Indicated	40.62 (38.72, 42.51)***	43.93 (42.02, 45.85)***	0.64 (0.61, 0.68)***	0.71 (0.21, 1.21)**	7.12 (5.30, 8.94)**	26.75 (-2.16, 55.65)
Diagnosed	42.96 (39.08, 46.83)**	37.18 (34.16, 40.20)***	0.70 (0.63, 0.78)***	0.36 (0.01, 0.70)	7.51 (4.15, 10.86)	55.37 (-7.61, 118.35)*

Statistical significance when comparing to mean of no condition denoted by ***p < 0.001, **p < 0.01, and *p < 0.05. Mean values calculated on weighted, imputed data (*N* = 6665). *HRQoL* is health-related quality of life, calculated using utility values obtained from responses to the EQ-5D-5L questionnaire

Compared with not having CHD, an indication of the condition and a diagnosis of it were each associated with more outpatient visits and higher OOP spending on healthcare, on average. Those diagnosed with CHD also had more inpatient admissions, on average. After adjustment, an indication of CHD was estimated to be associated with 2.1 (95% CI 0.8, 3.4) more outpatient visits per annum and $29.08 (95% CI − $1.49, $59.64) higher OOP spending per annum than the respective means for those without CHD. After adjustment, only mean inpatient admissions remained significantly higher for those with a CHD diagnosis than for those without the condition (0.18, 95% CI 0.03, 0.34), with no significant differences seen in outpatient visits and OOP spending.

An indication of hypertension and an indication of diabetes were both associated with lower mean PCS scores and HRQoL compared with not having the respective condition. After adjustment for covariates, these differences were not statistically significant, while diagnosed hypertension and diagnosed diabetes were associated with lower PCS scores (hypertension 3.15 95% CI 2.46, 3.84; diabetes 1.53 95% 0.86, 2.21) and HRQoL (hypertension 0.04 95% CI 0.03, 0.06; diabetes 0.02 95% CI, 0.01, 0.04), and with more outpatient visits (hypertension 2.63 95% CI 1.67, 3.60; diabetes 2.79 95% 1.75, 3.82) and OOP spending (hypertension $20.42 95% CI $8.59, $32.26; diabetes $25.10, 95% CI $11.15, $39.05), on average, compared with not having the respective conditions.

Mean PCS, MCS, and HRQoL scores were all significantly lower both for people with an indication of depression and for those diagnosed with depression than for those without depression. This was true without and with adjustment for covariates. Both unadjusted and adjusted estimates indicate that those with an indication of depression and those diagnosed with the condition had similar mean PCS and HRQoL scores. After adjustment, diagnosed depression was associated with a mean MCS score that was 12.58 points (95% CI 9.59, 15.58) lower than the mean for those with no depression, while those with an indication of depression also had a lower MCS score, though half as large in magnitude (6.70, 95% CI 5.43, 7.97).

Adding BMI as an additional covariate in the multivariate models had little impact on the magnitude and significance of the estimates of the partial associations of the outcomes with both an indication of each condition and its diagnosis (Additional file 1: Table S4). Complete case analysis yielded partial associations of similar direction, magnitude, and significance as those estimated using the multiply-imputed dataset (Additional file 1: Table S5).

## Discussion

In LMICs, high prevalence of undiagnosed chronic conditions [3–6, 12], particularly cardiovascular disease risk factors, has rightly aroused concern about an iceberg of NCD that could strain health systems ill-prepared to respond to a double burden of disease [46]. These concerns motivate a push for earlier diagnosis of chronic conditions, which could well be a health system priority. Assessment of the likely consequences of such a policy requires better evidence on the health and healthcare utilization of people with an indication but not a diagnosis of chronic conditions. If they have poor health and make heavy use of healthcare, then diagnosis, and consequent treatment, may bring immediate improvement in health while straining a health system less than would be the case if the newly diagnosed were previously light users of healthcare.

This study of a representative sample of the adult population of Sri Lanka revealed heterogeneity by type of chronic condition in the health and healthcare utilization of those with an indication but not a diagnosis of a condition. We found that having an indication but not a diagnosis of either CHD or depression—both symptomatic conditions—was associated with worse physical and mental health functioning and, for depression, worse HRQoL, after adjustment for age, ethnicity, education, sector and province of residence, wealth quintile, household size and composition, and the other chronic conditions of interest. Point estimates suggest that indications of these two conditions may also be associated with greater utilization of healthcare and higher OOP medical spending, although most of these estimates do not reach conventional levels of statistical significance after adjustment. In contrast, having an indication but not a diagnosis of either hypertension or diabetes—two conditions that can remain asymptomatic for some time—was not partially associated with worse health functioning and HRQoL, nor with higher healthcare utilization and OOP spending, after adjustment. These findings suggest that the health, healthcare, and economic burdens of undiagnosed chronic conditions may well depend, as would be logical, on the degree to which any condition is symptomatic. Those suffering symptoms of an undiagnosed condition may seek relief through medical treatment. Asymptomatic conditions would not be expected to induce the same loss of health and level of healthcare seeking.

We found that for each of CHD and depression, the likelihood of being diagnosed was lower than the likelihood of having an undiagnosed indication, while the opposite was true for hypertension and diabetes. This discrepancy may be partly due to the relatively higher cost and complexity of diagnosing the first two conditions. Health system constraints may slow the diagnosis of CHD and depression, which may partly explain the lower functioning and HRQoL of people with undiagnosed indications of these conditions. Less technology and skills are required to diagnose hypertension and diabetes, which may contribute to quicker diagnosis and less health impact among those not yet diagnosed.

We found that people with an indication of CHD had similar limitations in physical functioning and more limitations in mental functioning than people diagnosed with CHD, after adjustment. We estimated that an indication of CHD was associated with lower physical and mental functioning equal in magnitude to about 4–5% in the respective score. An indication of CHD was estimated to be associated with at least two outpatient visits per year more than the average with no indication of CHD after adjustment—a 46% increase. The absolute increase in the number of outpatient visits was similar to that associated with being diagnosed with CHD without adjustment. This is similar to findings in Indonesia, where people with undiagnosed “heart problems” had an additional 1.9 outpatient visits per year than those who did not have heart problems [22]. After adjusting for covariates, we found that OOP spending of those with an indication of CHD was almost 85% higher than those with no indication of CHD, on average. As a result of these increases, the utilization of outpatient care and the OOP spending of people with an indication of CHD were similar to the average levels of those with diagnosed CHD, hypertension, and diabetes. In Indonesia, people with undiagnosed heart problems did report higher outpatient and inpatient medical expenses, though this was not statistically significant, but they also reported higher expenditures on self-treatment [22]. The findings in Sri Lanka suggest that targeting people with an indication of CHD but not yet diagnosed should be prioritized given health and healthcare outcomes that are on par with people already diagnosed with CHD risk factors. For hypertension and diabetes, screening to identify people with indications of these conditions can still be worthwhile to reverse or slow progression to worse outcomes observed among those who are eventually diagnosed [47].

After adjusting for covariates, we found that the lower levels of physical functioning and HRQoL associated with an indication of depression were as large as the respective reductions associated with diagnosed depression. As would be expected, an indication of depression was associated with a reduction in mental functioning that was a little more than half the magnitude of the reduction associated with diagnosed depression. However, on average, those with an indication of depression scored 13% lower in mental functioning than those with no indication of depression after full adjustment. These findings suggest that people with symptoms of depression experience substantial losses of health and related quality of life that, with the exception of mental functioning, were similar to those experienced by those diagnosed with depression. There were similarities with findings in Japan, where people with undiagnosed depression had lower physical and mental functioning than those without depression, and similar to those with diagnosed depression [25]. The fact that reductions in health and quality of life associated with an indication of depression were substantially larger than those estimated for an indication of CHD gives further reason to increase efforts to identify Sri Lankans living with undiagnosed depression.

We found that indications of hypertension and diabetes were not associated with worse health and greater healthcare utilization, while these outcomes were associated with diagnosed hypertension and diabetes. These findings are consistent with evidence from Indonesia showing that outpatient use and OOP spending were higher for people with self-reported hypertension and diabetes, but these outcomes were not higher for people with undiagnosed hypertension and diabetes [22]. Our estimates are also consistent with other evidence that people with undiagnosed hypertension report better physical health than people diagnosed with the condition [24, 48]. There are several potential explanations for these consistencies. First, people who are undiagnosed may have had the condition for a shorter period and are less likely to be symptomatic, and so may not have experienced a loss of health which would also cause their demand for healthcare to increase [22]. Second, the association of diagnosed diabetes and hypertension with more outpatient visits and OOP spending is expected as these people should have had regular follow-up visits to manage their condition, while people with indications of diabetes and hypertension may not have sought additional healthcare as they had no perceived requirement. Lastly, among those with indications of hypertension and diabetes based on measurements taken in a single encounter, there are likely to be many false positives [22]. As with awareness–treatment–control studies of hypertension and diabetes, there is a risk of misclassifying individuals without these conditions as having an indication of them. This would dilute associations between true indications of hypertension or diabetes and health and healthcare outcomes.

There were several limitations in our study. Due to the nature of the field survey, we were limited to one measurement of biomarkers. The biomarkers used to define diabetes in this study were likely to be more precise than the symptomatic criteria used in a related study [22]. Furthermore, single biomarker measurements for hypertension and diabetes are commonly used in cascades of care studies to assess the performance of healthcare systems [4, 49].

To identify undiagnosed heart problems [22] and depression [25], we used methods that are commonly used to estimate the prevalence of these conditions. While an indication of a chronic condition is often used to identify undiagnosed cases [22, 24, 25], there is variation in the positive predictive values of the indicator tools. Nevertheless, our study suggests, at the very least, that people with symptoms that are indicative of CHD and depression were likely to experience poorer health and healthcare outcomes than those without indications of these conditions. We did not assess whether the duration since diagnosis was associated with worse outcomes.

The algorithm used to calculate physical and mental health functioning scores has been validated in several other countries [50] but not in Sri Lanka. For comparisons within a country, it is expected that the US-based algorithm will provide similar results to a country-derived one [51], although we cannot be sure of this.

## Conclusions

Undiagnosed people with indications of symptomatic conditions like CHD and depression are likely to have poorer health and use more healthcare than people without these conditions. Outcomes can even be worse for the undiagnosed than for the diagnosed. This suggests that management of people with indications of CHD and depression should be prioritized as the burden of these undiagnosed conditions was almost as high as it was for those diagnosed with these conditions. Getting these people diagnosed and onto effective disease management programmes that pay attention to follow-up and treatment compliance may not raise demands on health systems by so much since the undiagnosed are already heavy users of healthcare. In contrast, people with indications of typically asymptomatic conditions like hypertension and diabetes, show similar outcomes to those without these conditions. Here, diagnosis and effective management and control could still generate important health benefits by slowing disease progression.

## Supplementary Information

**Additional file 1:** Supplementary methods (Estimation of household SES using principal components analysis; Multiple imputation method) and Supplementary results (Complete case and imputed sample characteristics; Contingency table showing percentage of people with comorbid conditions; Adjusted differences in mean health and healthcare outcomes; AME controlling for BMI; and AME using complete case analysis).

## Data Availability

The data that support the findings of this study are available from the SLHAS Consortium but restrictions apply to the availability of these data, which were used under license for the current study, and so are not publicly available. The SLHAS Consortium, which has adopted an open data policy, will provide access to SLHAS Wave 1 data from 2024, on application to the Consortium by interested researchers. The specific data file used for this paper can be obtained from Nilmini Wijemunige upon reasonable request (nilmini@ihp.lk), and with permission of the SLHAS Consortium.

## Abbreviations

AME: Average marginal effects
BMI: Body mass index
CHD: Coronary heart disease
CI: Confidence interval
GLM: Generalized linear model
HRQoL: Health-related quality of life
LMICs: Low- and middle-income countries
MCS: Mental component summary
NCD: Non-communicable diseases
OLS: Ordinary least squares
OOP: Out-of-pocket
PCS: Physical component summary
PHQ: Patient Health Questionnaire
SD: Standard deviation
SES: Socioeconomic status
SF-12: 12-Item Short Form Survey
SLHAS: Sri Lanka Health and Ageing Study
