# Supplementary material for: Health outcomes and healthcare utilization associated with four undiagnosed chronic conditions: evidence from nationally representative survey data in Sri Lanka
Source: BMC Glob Public Health. 2024 Jul 8;2:45. doi: 10.1186/s44263-024-00075-0 (PMC11228003; doi:10.1186/s44263-024-00075-0)
Supplement: Supplementary file 1 — Additional file 1: Supplementary methods (Estimation of household SES using principal components analysis; Multiple imputation method) and Supplementary results (Complete case and imputed sample characteristics; Contingency table showing percentage of people with comorbid conditions; Adjusted differences in mean health and healthcare outcomes; AME controlling for BMI; and AME using complete case analysis). [file 44263_2024_75_MOESM1_ESM.docx]

# ADDITIONAL FILE 1

Health outcomes and healthcare utilization associated with four undiagnosed chronic conditions: Evidence from nationally representative survey data in Sri Lanka

*Nilmini Wijemunige, Pieter van Baal, Ravindra P. Rannan-Eliya, Owen O’Donnell*

Supplementary Methods 2

Estimation of household socioeconomic status using principal components analysis 2

Multiple imputation method 3

Supplementary Results 4

Table S1 Complete case and imputed sample characteristics 4

Table S2 Contingency table showing percentage (95% confidence interval) of people with comorbid conditions for each chronic condition state of CHD, hypertension, diabetes and depression 5

Table S3 Adjusted differences in mean health and healthcare outcomes between indication/diagnosis and absence of each chronic condition 6

Table S4 Average marginal effects of chronic condition states on outcome variables, controlling for BMI 7

Table S5 Average marginal effects of chronic condition states on outcome variables, using complete case analysis 8

Supplementary Methods

Estimation of household socioeconomic status using principal components analysis

The SLHAS Wave 1 uses an asset index approach to generate a proxy measure of each household’s living standard. The index was computed by using principal components analysis (PCA) of a set of household‑level variables relating to asset ownership or household characteristics. Variables were selected from those used in recent Sri Lanka Household Income and Expenditure Surveys conducted by the Department of Census and Statistics, selecting those with most predictive performance, and excluding some assets that are only relevant to agricultural households (e.g., tractor, thresher, fishing equipment). Variables were either dichotomous (e.g., household has a car) or categorical (e.g., type of drinking water source), apart from one ordinal variable (number of bedrooms). Dichotomous variables consisted of whether the household possessed each of the following items: radio/cassette player, television, VCD/DVD player, washing machine, fridge, electric fan, domestic phone, mobile phone, computer, internet access, camera/video camera, bicycle, motorcycle/scooter, three-wheeler, motor car/van, and bus/lorry/tipper.

Categorical variables were transformed into dichotomous indicators by creating separate dummy variables for each category. They consisted of the following (numbers in parentheses indicates number of categories in each): flooring material (5), material of wall (7), type of housing tenure (12), drinking water source (16), type of toilet (4), method of household garbage disposal (6), lighting power source (5), cooking fuel (13), and type of cooking place (3).

There was a small percentage of missing values in each variable (2–3%). These were imputed with either the PSU or stratum level mean of the variable or failing those the district/sector or national means. The principal component factor or index obtained by PCA after combining all these variables was then used to divide the sample into population weighted quantiles of equal size. Separate indices were not estimated for urban or rural sectors, but analysis indicates little difference between sectors in how the national index performs.

*Reproduced with permission from:*

Rannan-Eliya RP, Wijemunige N, Perera P, Kapuge Y, Gunawardana N, Sigera C, Jayatissa R, Herath HM, Gamage A, Weerawardena N, Sivagnanam I. Prevalence of diabetes and pre-diabetes in Sri Lanka: a new global hotspot–estimates from the Sri Lanka Health and Ageing Survey 2018/2019. BMJ Open Diabetes Research and Care. 2023 Feb 1;11(1):e003160.

Multiple imputation method

We used the *mi impute* family of functions in Stata to impute data. First, with registered variables with no missing values as “regular” variables: namely, participant age, sex, socioeconomic quintile, sector and district of residence, and ethnicity. Next, we registered variables of interest that had missing values as “imputed variables”, including inpatient and outpatient visits (annualized), recall period of inpatient and outpatient visits, out-of-pocket expenditures, household size, number of people in the household below 15 and above 60, body mass index, smoking status, physical component score, mental component score, utility value, diagnosis status of CHD, hypertension, diabetes and depression. Multiple imputation was performed with chained equations (*mi impute chained*) and predictive mean matching (*pmm*) using 10 nearest neighbors (*kn(10)).*

Supplementary Results

Table S1 Complete case and imputed sample characteristics

|  | **Complete case, unweighted (*N=*6,137)** | |  | **Imputed, unweighted (*N=*6,665)** | |
| --- | --- | --- | --- | --- | --- |
|  | **n / mean** | **% / SD** |  | **n / mean** | **% / SD** |
| Age, mean (SD) | 49.8 | 17.2 |  | 50.1 | 17.2 |
|  |  |  |  |  |  |
| Sex |  |  |  |  |  |
| Male | 3,019 | 49.2 |  | 3,268 | 49.0 |
| Female | 3,118 | 50.8 |  | 3,397 | 51.0 |
|  |  |  |  |  |  |
| Ethnicity |  |  |  |  |  |
| Sinhala | 4,324 | 70.5 |  | 4,707 | 70.6 |
| Tamil | 1,384 | 22.6 |  | 1,504 | 22.6 |
| Muslim | 403 | 6.6 |  | 428 | 6.4 |
| Other | 26 | 0.4 |  | 26 | 0.4 |
|  |  |  |  |  |  |
| Education |  |  |  |  |  |
| No formal schooling | 227 | 3.7 |  | 258 | 3.9 |
| Primary educated | 847 | 13.8 |  | 937 | 14.1 |
| Secondary educated | 4,812 | 78.4 |  | 5,199 | 78.0 |
| Tertiary educated | 251 | 4.1 |  | 272 | 4.1 |
|  |  |  |  |  |  |
| Sector |  |  |  |  |  |
| Urban | 1,840 | 30.0 |  | 2,024 | 30.4 |
| Rural | 3,395 | 55.3 |  | 3,661 | 54.9 |
| Estate | 165 | 2.7 |  | 170 | 2.6 |
| Rural/Estate | 737 | 12.0 |  | 810 | 12.2 |
|  |  |  |  |  |  |
| Province |  |  |  |  |  |
| Western | 1,284 | 20.9 |  | 1,435 | 21.5 |
| Central | 886 | 14.4 |  | 976 | 14.6 |
| Southern | 788 | 12.8 |  | 851 | 12.8 |
| Northern | 644 | 10.5 |  | 691 | 10.4 |
| Eastern | 527 | 8.6 |  | 553 | 8.3 |
| North-Western | 523 | 8.5 |  | 548 | 8.2 |
| North-Central | 446 | 7.3 |  | 477 | 7.2 |
| Uva | 433 | 7.1 |  | 467 | 7.0 |
| Sabaragamuwa | 606 | 9.9 |  | 667 | 10.0 |
|  |  |  |  |  |  |
| SES quintile |  |  |  |  |  |
| Poorest | 1,457 | 23.7 |  | 1,568 | 23.5 |
| Poorer | 1,221 | 19.9 |  | 1,328 | 19.9 |
| Middle | 1,131 | 18.4 |  | 1,245 | 18.7 |
| Richer | 1,110 | 18.1 |  | 1,220 | 18.3 |
| Richest | 1,218 | 19.8 |  | 1,304 | 19.6 |
|  |  |  |  |  |  |
| Household size, mean (SD) | 2.98 | 1.4 |  | 2.98 | 1.4 |
| Proportion below 15, mean (SD) | 0.07 | 0.16 |  | 0.07 | 0.16 |
| Proportion above 60, mean (SD) | 0.22 | 0.33 |  | 0.23 | 0.33 |
|  |  |  |  |  |  |

Table S2 Contingency table showing percentage (95% confidence interval) of people with comorbid conditions for each chronic condition state of CHD, hypertension, diabetes and depression

|  | **Comorbid condition, % (95% CI)** | | | | | | |
| --- | --- | --- | --- | --- | --- | --- | --- |
|  | **CHD** |  | **Hypertension** |  | **Diabetes** |  | **Depression** |
| CHD | - |  | 52.0 (47.0, 57.1) |  | 32.7 (27.6, 37.7) |  | 12.1 (8.9, 15.3) |
|  |  |  |  |  |  |  |  |
| Hypertension | 16.9 (15.1, 18.7) |  | - |  | 37.6 (34.8, 40.5) |  | 7.4 (6.1, 8.7) |
|  |  |  |  |  |  |  |  |
| Diabetes | 15.1 (12.9, 17.4) |  | 53.8 (50.3, 57.3) |  | - |  | 8.2 (6.4, 9.9) |
|  |  |  |  |  |  |  |  |
| Depression | 22.3 (16.6, 27.9) |  | 42.2 (35.0, 49.4) |  | 32.5 (25.7, 39.4) |  | - |
|  |  |  |  |  |  |  |  |
| Full sample | 9.6 (8.7, 10.6) |  | 29.7 (28.1, 31.3) |  | 20.8 (19.4, 22.2) |  | 5.2 (4.5, 5.9) |
|  |  |  |  |  |  |  |  |

*Notes:* Analysis on weighted, imputed data (*N=*6,665). 16.9% of people with hypertension have CHD, while 52.0% of people with CHD have hypertension. There are slight variations compared to prevalence estimates published individually for hypertension and diabetes given methodological differences in weighting for the full sample and use of imputed data.

Table S3 Adjusted differences in mean health and healthcare outcomes between indication/diagnosis and absence of each chronic condition

|  | **Health functioning (SF-12)** | | | | | |  | **HRQoL (95% CI)** |  |  | **Inpatient visits (95% CI)** |  |  | | **Outpatient visits (95% CI)** |  |  | **OOP spending, USD (95% CI)** |  |
| --- | --- | --- | --- | --- | --- | --- | --- | --- | --- | --- | --- | --- | --- | --- | --- | --- | --- | --- | --- |
|  | **Physical (95% CI)** |  |  | **Mental (95% CI)** |  |  | |  |  |  |  |  | |  |  |  |  |  |  |
| CHD (Ref: No condition) | |  |  |  |  |  | |  |  |  |  |  | |  |  |  |  |  |  |
| Indicated | -2.65 (-3.66, -1.63) | *** |  | -2.25 (-3.38, -1.12) | *** |  | | -0.01 (-0.03, 0.01) |  |  | 0.11 (-0.02, 0.23) |  | |  | 2.13 (0.81, 3.44) | ** |  | 29.08 (-1.49, 59.64) |  |
| Diagnosed | -2.66 (-3.82, -1.49) | *** |  | -0.55 (-1.68, 0.58) |  |  | | -0.02 (-0.04, 0.00) | * |  | 0.18 (0.03, 0.34) | * | |  | -0.09 (-1.19, 1.02) |  |  | -2.87 (-11.80, 6.07) |  |
|  |  |  |  |  |  |  | |  |  |  |  |  | |  |  |  |  |  |  |
| Hypertension (Ref: No condition) | |  |  |  |  |  | |  |  |  |  |  | |  |  |  |  |  |  |
| Indicated | -0.05 (-0.66, 0.55) |  |  | -0.49 (-1.15, 0.16) |  |  | | 0.00 (-0.01, 0.01) |  |  | -0.04 (-0.13, 0.05) |  | |  | -0.24 (-1.00, 0.52) |  |  | 5.10 (-3.03, 13.23) |  |
| Diagnosed | -3.15 (-3.84, -2.46) | *** |  | -0.24 (-0.93, 0.46) |  |  | | -0.04 (-0.06, -0.03) | *** |  | 0.09 (-0.02, 0.21) |  | |  | 2.63 (1.67, 3.60) | *** |  | 20.42 (8.59, 32.26) | ** |
|  |  |  |  |  |  |  | |  |  |  |  |  | |  |  |  |  |  |  |
| Diabetes (Ref: No condition) | |  |  |  |  |  | |  |  |  |  |  | |  |  |  |  |  |  |
| Indicated | 0.11 (-0.66, 0.88) |  |  | 0.38 (-0.50, 1.25) |  |  | | 0.00 (-0.02, 0.01) |  |  | -0.04 (-0.14, 0.06) |  | |  | -0.03 (-0.99, 0.94) |  |  | -3.11 (-12.17, 5.95) |  |
| Diagnosed | -1.53 (-2.21, -0.86) | *** |  | -0.29 (-0.98, 0.41) |  |  | | -0.02 (-0.04, -0.01) | ** |  | 0.13 (-0.01, 0.27) |  | |  | 2.79 (1.75, 3.82) | *** |  | 25.10 (11.15, 39.05) | *** |
|  |  |  |  |  |  |  | |  |  |  |  |  | |  |  |  |  |  |  |
| Depression (Ref: No condition) | |  |  |  |  |  | |  |  |  |  |  | |  |  |  |  |  |  |
| Indicated | -5.78 (-6.91, -4.64) | *** |  | -6.70 (-7.97, -5.43) | *** |  | | -0.18 (-0.20, -0.16) | *** |  | 0.21 (-0.01, 0.42) |  | |  | 1.20 (-0.16, 2.57) |  |  | -0.96 (-14.43, 12.51) |  |
| Diagnosed | -4.79 (-7.30, -2.27) | *** |  | -12.58 (-15.58, -9.59) | *** |  | | -0.11 (-0.16, -0.07) | *** |  | 0.44 (-0.16, 1.03) |  | |  | 3.99 (0.27, 7.71) | * |  | 15.43 (-17.39, 48.24) |  |
|  |  |  |  |  |  |  | |  |  |  |  |  | |  |  |  |  |  |  |

*Notes:* Each column shows an outcome of interest. These models control for demographic features – age, sex, ethnicity, education, sector, province, socioeconomic quintile, household composition – and each chronic condition state (no condition, indication of condition, diagnosed condition). The statistical significance of the AME compared to no condition (indication vs no condition; diagnosed vs no condition) is denoted by *** p<0.001, ** p<0.01, * p<0.05. Analyses is on unweighted, imputed data (*N=*6,665*)*. HRQoL is health‑related quality of life, calculated using utility values obtained from responses to the EQ-5D-5L questionnaire.

Table S4 Average marginal effects of chronic condition states on outcome variables, controlling for BMI

|  | **Health functioning (SF-12)** | | | | |  | **HRQoL (95% CI)** |  |  | **Inpatient visits (95% CI)** |  |  | **Outpatient visits (95% CI)** |  |  | **OOP spending, USD (95% CI)** |  |
| --- | --- | --- | --- | --- | --- | --- | --- | --- | --- | --- | --- | --- | --- | --- | --- | --- | --- |
|  | **Physical (95% CI)** |  |  | **Mental (95% CI)** |  |  |  |  |  |  |  |  |  |  |  |  |  |
|  | |  |  |  |  |  |  |  |  |  |  |  |  |  |  |  |  |
| CHD (Ref: No condition) | |  |  |  |  |  |  |  |  |  |  |  |  |  |  |  |  |
| Indication | -2.69 (-3.71, -1.67) | *** |  | -2.24 (-3.37, -1.11) | *** |  | -0.01 (-0.03, 0.01) |  |  | 0.11 (-0.02, 0.23) |  |  | 2.13 (0.81, 3.45) | ** |  | 29.81 (-0.41, 60.03) |  |
| Diagnosed | -2.63 (-3.79, -1.47) | *** |  | -0.55 (-1.69, 0.58) |  |  | -0.02 (-0.04, 0.00) |  |  | 0.19 (0.03, 0.34) | * |  | -0.07 (-1.17, 1.04) |  |  | -3.12 (-12.00, 5.75) |  |
|  |  |  |  |  |  |  |  |  |  |  |  |  |  |  |  |  |  |
| Hypertension (Ref: No condition) | |  |  |  |  |  |  |  |  |  |  |  |  |  |  |  |  |
| Indication | 0.10 (-0.51, 0.70) |  |  | -0.54 (-1.20, 0.12) |  |  | 0.01 (-0.01, 0.02) |  |  | -0.04 (-0.13, 0.05) |  |  | -0.25 (-1.01, 0.51) |  |  | 4.21 (-3.83, 12.24) |  |
| Diagnosed | -2.98 (-3.67, -2.29) | *** |  | -0.29 (-0.98, 0.41) |  |  | -0.04 (-0.05, -0.02) | *** |  | 0.10 (-0.02, 0.21) |  |  | 2.61 (1.64, 3.58) | *** |  | 18.49 (7.65, 29.34) | ** |
|  |  |  |  |  |  |  |  |  |  |  |  |  |  |  |  |  |  |
| Diabetes (Ref: No condition) | |  |  |  |  |  |  |  |  |  |  |  |  |  |  |  |  |
| Indication | 0.27 (-0.51, 1.05) |  |  | 0.33 (-0.55, 1.21) |  |  | 0.00 (-0.01, 0.02) |  |  | -0.03 (-0.14, 0.07) |  |  | -0.01 (-0.98, 0.96) |  |  | -3.99 (-12.69, 4.70) |  |
| Diagnosed | -1.54 (-2.21, -0.86) | *** |  | -0.28 (-0.97, 0.41) |  |  | -0.02 (-0.03, -0.01) | ** |  | 0.14 (-0.01, 0.28) |  |  | 2.82 (1.78, 3.85) | *** |  | 24.94 (11.35, 38.52) | *** |
|  |  |  |  |  |  |  |  |  |  |  |  |  |  |  |  |  |  |
| Depression (Ref: No condition) | |  |  |  |  |  |  |  |  |  |  |  |  |  |  |  |  |
| Indication | -5.75 (-6.89, -4.62) | *** |  | -6.71 (-7.98, -5.44) | *** |  | -0.18 (-0.20, -0.16) | *** |  | 0.20 (-0.01, 0.42) |  |  | 1.18 (-0.18, 2.54) |  |  | -1.37 (-14.17, 11.43) |  |
| Diagnosed | -4.78 (-7.28, -2.27) | *** |  | -12.58 (-15.58, -9.59) | *** |  | -0.11 (-0.16, -0.07) | *** |  | 0.42 (-0.17, 1.01) |  |  | 4.10 (0.34, 7.86) | * |  | 14.83 (-16.95, 46.61) |  |
|  |  |  |  |  |  |  |  |  |  |  |  |  |  |  |  |  |  |
| BMI category (Ref: Normal) | |  |  |  |  |  |  |  |  |  |  |  |  |  |  |  |  |
| Overweight | -0.55 (-1.04, -0.05) | * |  | 0.13 (-0.40, 0.66) |  |  | -0.02 (-0.03, -0.01) | ** |  | -0.04 (-0.12, 0.04) |  |  | -0.25 (-0.92, 0.41) |  |  | 9.63 (0.93, 18.32) | * |
| Obese | -1.93 (-2.70, -1.16) | *** |  | 0.62 (-0.21, 1.45) |  |  | -0.06 (-0.08, -0.05) | *** |  | 0.02 (-0.12, 0.17) |  |  | 0.48 (-0.64, 1.61) |  |  | 13.17 (-0.53, 26.86) |  |

*Notes:* Each column shows an outcome of interest. These models use the same covariates as in Table S3, with additional control for body mass index (BMI) categories (normal <25 kg/m^2^, overweight 25-29.9 kg/m^2^, obese ≥ 30 kg/m^2^). The statistical significance of the AME compared to no condition (indication vs no condition; diagnosed vs no condition) is denoted by *** p<0.001, ** p<0.01, * p<0.05. Analyses is on unweighted, imputed data (*N=*6,665). HRQoL is health‑related quality of life, calculated using utility values obtained from responses to the EQ-5D-5L questionnaire.

Table S5 Average marginal effects of chronic condition states on outcome variables, using complete case analysis

|  | **Health functioning (SF-12)** | | | |  | | **HRQoL (95% CI)** |  |  | **Inpatient visits (95% CI)** |  |  | **Outpatient visits (95% CI)** |  |  | **OOP spending,  USD (95% CI)** |  |
| --- | --- | --- | --- | --- | --- | --- | --- | --- | --- | --- | --- | --- | --- | --- | --- | --- | --- |
|  | **Physical (95% CI)** |  |  | **Mental (95% CI)** |  |  |  |  |  |  |  |  |  |  |  |  |  |
|  | *N = 6,313* |  |  | *N = 6,313* |  |  | *N = 6,350* |  |  | *N = 6,411* |  |  | *N = 6,432* |  |  | *N = 6,443* |  |
|  | |  |  |  |  |  |  |  |  |  |  |  |  |  |  |  |  |
| CHD (Ref: No condition) | |  |  |  |  |  |  |  |  |  |  |  |  |  |  |  |  |
| Indication | -2.67 (-3.69, -1.65) | *** |  | -2.35 (-3.49, -1.21) | *** |  | -0.01 (-0.03, 0.01) |  |  | 0.11 (-0.01, 0.23) |  |  | 2.16 (0.83, 3.48) | ** |  | 30.26 (-0.95, 61.46) |  |
| Diagnosed | -2.55 (-3.71, -1.39) | *** |  | -0.44 (-1.58, 0.70) |  |  | -0.02 (-0.04, 0.00) | * |  | 0.18 (0.03, 0.32) | * |  | -0.11 (-1.22, 1.00) |  |  | -3.02 (-11.92, 5.89) |  |
|  |  |  |  |  |  |  |  |  |  |  |  |  |  |  |  |  |  |
| Hypertension (Ref: No condition) | |  |  |  |  |  |  |  |  |  |  |  |  |  |  |  |  |
| Indication | -0.03 (-0.64, 0.59) |  |  | -0.56 (-1.22, 0.09) |  |  | 0.00 (-0.01, 0.01) |  |  | -0.04 (-0.13, 0.05) |  |  | -0.31 (-1.06, 0.44) |  |  | 4.78 (-3.02, 12.58) |  |
| Diagnosed | -3.21 (-3.90, -2.52) | *** |  | -0.33 (-1.02, 0.37) |  |  | -0.04 (-0.06, -0.03) | *** |  | 0.10 (-0.02, 0.21) |  |  | 2.63 (1.66, 3.59) | *** |  | 19.99 (8.45, 31.52) | *** |
|  |  |  |  |  |  |  |  |  |  |  |  |  |  |  |  |  |  |
| Diabetes (Ref: No condition) | |  |  |  |  |  |  |  |  |  |  |  |  |  |  |  |  |
| Indication | 0.14 (-0.62, 0.90) |  |  | 0.42 (-0.46, 1.29) |  |  | 0.00 (-0.02, 0.02) |  |  | -0.04 (-0.14, 0.06) |  |  | -0.10 (-1.04, 0.83) |  |  | -3.24 (-12.30, 5.82) |  |
| Diagnosed | -1.54 (-2.22, -0.86) | *** |  | -0.24 (-0.94, 0.45) |  |  | -0.02 (-0.04, -0.01) | ** |  | 0.13 (-0.01, 0.27) |  |  | 2.76 (1.73, 3.79) | *** |  | 25.25 (11.10, 39.39) | *** |
|  |  |  |  |  |  |  |  |  |  |  |  |  |  |  |  |  |  |
| Depression (Ref: No condition) | |  |  |  |  |  |  |  |  |  |  |  |  |  |  |  |  |
| Indication | -5.74 (-6.88, -4.60) | *** |  | -6.77 (-8.03, -5.51) | *** |  | -0.18 (-0.21, -0.15) | *** |  | 0.21 (0.00, 0.42) |  |  | 1.15 (-0.21, 2.52) |  |  | -1.70 (-13.56, 10.17) |  |
| Diagnosed | -4.63 (-7.11, -2.15) | *** |  | -12.62 (-15.59, -9.65) | *** |  | -0.11 (-0.15, -0.07) | *** |  | 0.44 (-0.16, 1.05) |  |  | 3.93 (0.22, 7.65) | * |  | 15.49 (-17.58, 48.56) |  |

*Notes:* Each column shows an outcome of interest. These models use the same covariates as Table S3, however analyses is on (unweighted) **non-imputed** data*.* The statistical significance of the AME compared to no condition (indication vs no condition; diagnosed vs no condition) is denoted by *** p<0.001, ** p<0.01, * p<0.05. HRQoL is health‑related quality of life, calculated using utility values obtained from responses to the EQ-5D-5L questionnaire.
